# PED: a novel predictor-encoder-decoder model for Alzheimer drug molecular generation

**DOI:** 10.3389/frai.2024.1374148

**Published:** 2024-04-16

**Authors:** Dayan Liu, Tao Song, Kang Na, Shudong Wang

**Affiliations:** ^1^College of Computer Science and Technology, China University of Petroleum (East China), Qingdao, China; ^2^The Ninth Department of Health Care Administration, The Second Medical Center, Chinese PLA General Hospital, Beijing, China

**Keywords:** molecular generation, Alzheimer, deep learning, neural networks, drug design

## Abstract

Alzheimer's disease (AD) is a gradually advancing neurodegenerative disorder characterized by a concealed onset. Acetylcholinesterase (AChE) is an efficient hydrolase that catalyzes the hydrolysis of acetylcholine (ACh), which regulates the concentration of ACh at synapses and then terminates ACh-mediated neurotransmission. There are inhibitors to inhibit the activity of AChE currently, but its side effects are inevitable. In various application fields where Al have gained prominence, neural network-based models for molecular design have recently emerged and demonstrate encouraging outcomes. However, in the conditional molecular generation task, most of the current generation models need additional optimization algorithms to generate molecules with intended properties which make molecular generation inefficient. Consequently, we introduce a cognitive-conditional molecular design model, termed PED, which leverages the variational auto-encoder. Its primary function is to adeptly produce a molecular library tailored for specific properties. From this library, we can then identify molecules that inhibit AChE activity without adverse effects. These molecules serve as lead compounds, hastening AD treatment and concurrently enhancing the AI's cognitive abilities. In this study, we aim to fine-tune a VAE model pre-trained on the ZINC database using active compounds of AChE collected from Binding DB. Different from other molecular generation models, the PED can simultaneously perform both property prediction and molecule generation, consequently, it can generate molecules with intended properties without additional optimization process. Experiments of evaluation show that proposed model performs better than other methods benchmarked on the same data sets. The results indicated that the model learns a good representation of potential chemical space, it can well generate molecules with intended properties. Extensive experiments on benchmark datasets confirmed PED's efficiency and efficacy. Furthermore, we also verified the binding ability of molecules to AChE through molecular docking. The results showed that our molecular generation system for AD shows excellent cognitive capacities, the molecules within the molecular library could bind well to AChE and inhibit its activity, thus preventing the hydrolysis of ACh.

## 1 Introduction

Alzheimer's disease (AD) is a neurodegenerative condition that progresses subtly from its onset (Cummings and Cole, 2002). Symptoms in the clinical setting encompass memory deterioration, speech difficulties, apraxia, agnosia, deficits in visual-spatial abilities, executive function disturbances, and shifts in personality and behavior, etc. The etiology is unknown so far. AD has the characteristics of long course of disease, many causes and complicated pathology. There are other irregularities of neurotransmitters in the center in addition to the drop-in acetylcholine levels in the brain. Additionally, the aggregation of A, the disturbance of metal ion metabolism, the imbalance of calcium balance, the rise in free radicals, and the onset of inflammation are the primary causes of AD. In view of the above causes, the therapeutic targets of AD mainly include acetylcholinesterase (AChE), metal ions, Beta Amyloid Peptide (β-AP), monoamine oxidase (MAO), free radicals, tau protein, N-methyl-D-aspartate (NMDA) receptor and other related targets (Casal et al., 2002; Sambamurti et al., 2011). Acetylcholine (ACh) is the first neurotransmitter discovered by human beings, and its mediated neurotransmission is the basis of nervous system function. Sudden interruption of ACH-mediated neurotransmission is fatal, and its gradual loss is associated with progressive deterioration of cognitive, autonomic and neuromuscular functions (Klinkenberg et al., 2011). However, AChE is an efficient hydrolase that catalyzes the hydrolysis of ACh, which regulates the concentration of ACh at synapses and then terminates ACh-mediated neurotransmission. There are inhibitors to inhibit the activity of AChE, but its side effects are inevitable (Alonso et al., 2005) Therefore, there is an increasing demand for developing active compounds with stronger inhibitory function and minimal side effects. Artificial intelligence (AI) leverages acquired knowledge and insights to formulate decisions and strategize subsequent actions. Modern methods incorporate a variety of strategies relevant to areas such as decision-making or cognitive-enhanced network security. Given that modern machines often lack intuition, emotional intelligence, common sense, and other human-centric attributes essential for effective planning and decision-making, there's potential to enhance planning-focused cognitive technology through broader artificial intelligence research (Fintz et al., 2022; Liu et al., 2022; Qiu et al., 2022).

To efficiently generate molecule library with intended properties, we propose a cognitive conditional molecular design model based on VAE which can predict properties and generate molecules concurrently, named PED, to screen molecules that can inhibit AChE activity from the generated molecular libraries as lead compounds and accelerate the treatment of AD. In this study, we aim to finetune a VAE pre-trained on the ZINC database using active compounds of AChE collected from BindingDB. On the same data sets, PED performs better than other methods. Meanwhile, we show that the model can well generate specified molecular properties. Furthermore, we also verified the binding ability of molecules to AChE through molecular docking. The results showed that the molecules in the molecular library could bind to AChE well.

The main contributions of this manuscript are summarized as below.

1. We put forth a novel deep learning model based on variational auto-encoder, namely PED, to efficiently generate molecular library with desired properties for AChE, simultaneously, show the cognitive capacities of AI. PED is engineered to manage both property forecasting and molecular generation in tandem, striving for superior outcomes relative to advanced methods.

2. In PED, given a specific set of properties, it samples new molecules directly from the conditionally generated distribution without adding additional optimization processes like other models.

3. Extensive testing was carried out on the ZINC database to assess PED's efficacy. The outcomes from these tests highlighted PED's predominant performance over other deep generative frameworks.

4. Using active AChE compounds sourced from Binding DB, we refine the PED initially pre-trained on the ZINC database to produce a molecular library. Furthermore, we also verified the binding ability of molecules to AChE through molecular docking. The results showed that the molecules in the molecular library could bind to AChE well.

The subsequent sections of this research are structured in the following manner. Some previous studies in de novo molecular design are reviewed in Section 2. Our model is introduced in Section 3. Experimental results and conclusions are presented in Section 4. Performance Analysis and Section 5. Conclusion and the future work respectively.

## 2 Related works

In this section, we first review the development of deep learning in molecular generation in recent years, and then introduce several commonly used molecular generation strategies and models.

### 2.1 Deep learning in molecular generation

Within the realm of molecular design, virtual screening (VS) has conventionally been employed to pinpoint molecules potentially yielding optimal experimental outcomes (Shoichet, 2004). Contrasting de novo molecular design, the source of molecules is distinctive: in virtual screening, the structure is known in advance, while in molecular de novo design, it is an attempt to generate the structure to be evaluated. Although virtual screening libraries have become very large according to the standards of drug discovery, the chemical space corresponding to these libraries only occupies a small part. When considering such a compound library, the evaluation method may inevitably sacrifice the accuracy of prediction. By using de novo molecular design to generate molecules in a directional way, computational workers hope to cross the chemical space more effectively and obtain the best chemical solution while analyzing fewer molecules than large chemical libraries. In addition, for a given target, there may be many acceptable regions in chemical space. Hence, the objective of the molecular design approach is to strike a balance between exploring global solutions and harnessing local minima (Schneider, 2010; Müller et al., 2022).

Recently, with the advancement of artificial intelligence (AI), new practical experience has been gained in the field of drug discovery (Ding et al., 2017, 2022; Chu et al., 2022). In typical data domains like computer vision (Voulodimos et al., 2018; Borhani et al., 2022) and natural language processing (NLP) (Chowdhary, 2020; Ferruz et al., 2022), deep generative models have significantly advanced in representing data distributions (Meyers et al., 2021). Such techniques are also employed to mimic molecular distributions, understand the probabilistic distributions of vast molecule sets, and produce novel molecules by drawing samples from these distributions (Dauparas et al., 2022). In the realm of molecular structure generation, a variety of deep learning models have been suggested by scholars. These encompass techniques such as generative adversarial networks (GANs), variational autoencoders (VAEs), and recurrent neural systems (RNNs) (Creswell et al., 2018; Korshunova et al., 2022). In these methods, a molecule is represented as a simplified molecular-input line-entry system (SMILES) (Weininger, 1988). Most of the current molecular generation models are based on conditional molecular design and finally generate new molecules with properties close to the predetermined target conditions (Xu et al., 2019; Walters and Barzilay, 2020).

### 2.2 Molecular generation model

The descriptors of SMILES are generally implemented by using long-term and short-term memory networks (LSTM). Serving as a unique temporal cycle neural network, LSTM was crafted explicitly to tackle the pervasive issue of long-term dependencies inherent in traditional RNNs. Due to the characteristics of this cyclic algorithm, the cyclic structure and chiral center of molecules expressed by SMILES are presented more perfectly. LSTM can be used to generate molecular sets with or without filters. Grisoni et al. suggested the use of bidirectional generative RNNs for designing molecules based on SMILES. In pursuit of this, they employed two proven bidirectional approaches and pioneered a novel technique for augmenting data and generating SMILES strings, termed as bidirectional molecule design by alternate learning (BIMODAL) (Grisoni et al., 2020). In addition, Li et al. studied the ability of RNN-based de novo molecular design method to produce new molecular inhibitors in the research field of chemical space (Li et al., 2020). In their quest to formulate novel inhibitors for proto-oncogene serine/threonine protein kinase 1 (PIM1) and CDK4 kinase, they evaluated four compounds. Their efforts culminated in the identification of a potent PIM1 inhibitor and two primary compounds that hinder CDK4 activity.

There is also a class of deep learning algorithms for automatic encoders, such as VAE and adversarial auto-encoder (AAE), which use the description method of molecules in latent space to generate molecules. On the one hand, the molecular features of the training set are stored in latent space by encoder, and on the other hand, these molecular features are reconstituted into new molecules by decoder. Owing to this approach's utilization of continuous latent space accumulation, the newly generated molecular set retains the physicochemical property distribution inherent in the training set. Many models have been proposed that employ reasonable substructures as building blocks for generating high-quality molecules. Previous studies introduced a model termed chemical-vae (Gómez-Bombarelli et al., 2018), designed to produce novel molecules, enabling effective exploration and refinement within expansive chemical compound spaces. In order to generate effective molecular graphs, MHG-VAE proposed molecular hypergraph grammar (MHG) to encode chemical constraints (Kajino, 2019). The authors have proposed a reaction model to forecast the interaction among reactants, resulting in the creation of novel molecules. In lieu of VAE, the objective function incorporates minimization to acquire model parameters.

Another popular deep learning algorithm is GAN. The algorithm uses two functions, the generator and the discriminator, against each other to generate the desired molecules. Because of the discontinuity of the atoms that make up the molecule, the discriminator can't directly feedback the information to the generator. Referring to the method adopted in NLP, the information feedback is realized by a reward function or policy gradient. The reward equation serves as a filtering criterion. It not only preserves the property distribution of the generated set akin to the training set but also nudges the property distribution of the created set to shift toward a different direction. This algorithm can use various molecular description methods, such as SMILES, Latent space, or graph, and can meet various requirements by combining various screening conditions, so it is a potential algorithm. Prykhodko et al. introduced LatentGAN, a novel deep learning framework that integrates an autoencoder with a generative adversarial neural network, tailored for de novo molecular design (Prykhodko et al., 2019).

To refine a sequence-based generative model specifically for molecular de novo design, Marcus and team formulated a technique capable of learning to construct structures with predetermined desirable characteristics, employing enhanced episodic likelihood (Olivecrona et al., 2017). M Popova et al. proposed a unique computational approach for the de novo design of molecules with targeted characteristics, named ReLeaSE, which utilizes deep learning and reinforcement learning methodologies (Popova et al., 2018). These methods used additional optimization processes instead of directly generating molecules of intended properties, which becomes inefficient.

#### 2.2.1 Atom-based molecular generation

Numerous atom-centric generative models employ SMILES for depicting molecules. Given that SMILES serves as a text-centric representation, chemistry generation methodologies can leverage sequence-appropriate deep learning structures like RNNs. By extensively pre-training on vast molecular structure datasets, the emergent model gains inherent knowledge, encapsulating the effective nuances of SMILES grammar and syntax. Initial endeavors leveraged transfer learning to skew generation toward desired chemical spaces. The prevalent approach now integrates generative tasks with RL algorithms, striving to attain higher rewards by discovering optimal molecules within the search landscape. Beyond the realm of SMILES-centric models, there's a growing fascination with models directly interpreting the topographical configurations of molecular graphs, where atoms and connections represent nodes and edges respectively. These graph-informed models aim to sidestep the synthetic facets of SMILES notation, offering a more innate depiction of molecular frameworks.GraphVAE and MolGAN are based on the method of generating graphs (De Cao and Kipf, 2018; Simonovsky and Komodakis, 2018), which can learn to generate the adjacency matrix of the whole graph at one time. Others describe the method of learning to generate molecules step by step by iteratively modifying molecular graphs. Recently, the RL method has shown promising results in the settings of the diagram.

#### 2.2.2 Fragment-based molecular generation

While atom-based generative models with prior training exhibit a strong inherent capability toward substructures present in their training sets, they retain the ability to adjust each molecular atom individually. Such adaptability enhances the model's expressiveness, thereby broadening its reach across the chemical space. Conversely, the fragment-based methodology employs a more generalized molecular depiction to constrain the exploration domain. Jin and his colleagues elucidated the workings of JTVAE, a dual-phase generation procedure (Jin et al., 2018). Initially, a nodal tree is developed to mirror the assembly of molecular subcomponents (resembling a simplification graph). Subsequently, a network transmitting graph information deciphers the ultimate molecular form. DeepFMPO, by weighing fragment resemblances in its optimization, attains superior efficacy (Al Jumaily et al., 2022).

Based on the above research, we propose a conditional molecular design model based on VAE, named PED, to efficiently generate a molecule library with intended properties and screen molecules that can inhibit AChE activity without negative consequences from the library as lead compounds, aiming to accelerate the treatment of AD. Unlike previous studies, the PED can perform property prediction and molecule generation simultaneously, which means that it can generate molecules with intended properties without additional optimization processes. This model can improve the efficiency of molecules generation while ensuring the quality of generated molecules.

## 3 Materials and methods

In this section, we first introduce the proposed variational auto-Encoder model for de novo molecular design, named PED. PED consists of three modules, namely predictor, encoder and decoder, by doing so, the model can predict the properties while generating molecules without additional optimization, aiming to ensure the efficiency of molecular generation. Finally, we introduce training procedure and evaluation metrics.

### 3.1 Model overview

The model consists of three 250-dimensional gated recurrent unit (GRU) networks: the predictor network, the encoder network and the decoder network. The predictor and encoder are made up of bidirectional GRUs network, while the decoder is a unidirectional GRU.

**Figure 1:**
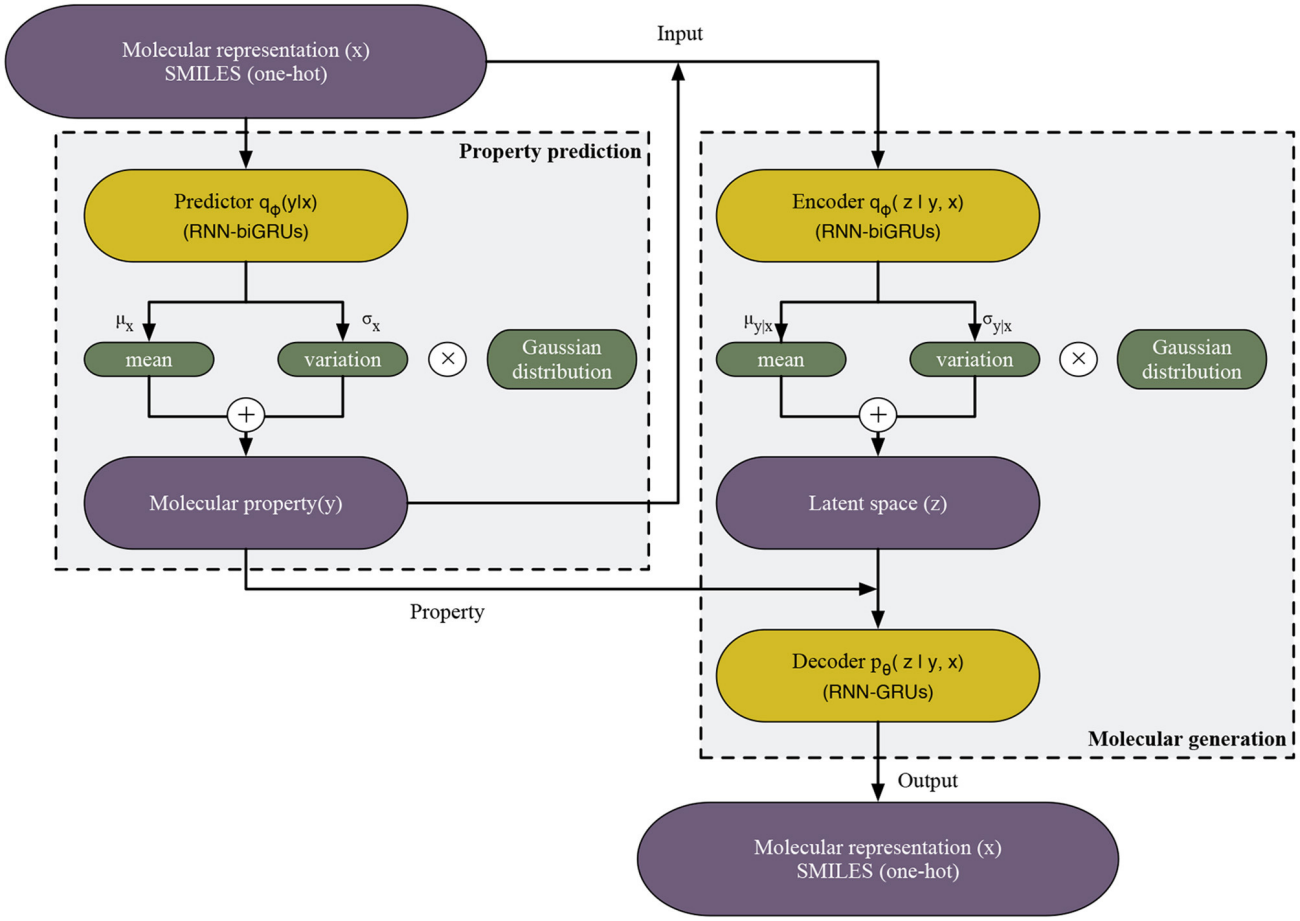
The workflow of PED model. Gray and blue areas separate PED into two components: (1) Property prediction, the labeled data are used for building a property predictor which introduces the gaussian distribution in order to address the intractability of y and (2) molecular generation, the autoencoder are trained by the labeled data. The RNN encoder maps x and y to the latent space z, similarly introducing the gaussian distribution, and then RNN decoder maps y and z to original x.

In order to forecast the subsequent character within the SMILES strings that depict molecular structures, the final layer incorporates a dense output layer coupled with a neuron unit that utilizes a softmax activation function. In this context, the synthesis of donepezil is showcased as a case study. Principally prescribed for Alzheimer's management, donepezil can be represented by the SMILES notation: “O=C(C(C=C(OC)C(OC)=C1)=C1C2)C2CC(CC3)CCN3CC4=CC=CC=C4”. The initial data for the system comprises a “one-hot” delineation of a SMILES string, whereby each string undergoes segmentation into various tokens. Here, the inaugural token is “O”, transformed into a “one-hot” vector and fed into the linguistic model. Subsequently, the model revises its concealed state and forecasts the probability spread over forthcoming viable tokens, decoded as “=” in this instance. Supplying the one-hot representation of “=” prompts the model to modify its concealed state during the forthcoming cycle, leading to the revelation of the succeeding token. This recurrent process, tackling one token at a time, persists until the “\n” character surfaces, signifying the culmination of the SMILES sequence, thus generating the final SMILES notation for donepezil (Figure 1).

In the case of the previously delineated predictor and encoder networks, we introduce distinct fixed-form distributions. Specifically, we define q_ϕ_(y ∣ x) and q_ϕ_(*z* ∣ y, x), each parametrized by ϕ. These distributions aim to approximate the true posterior distribution, employing a widely employed method in efficient variational inference, as described in Equations (1, 2):


(1) $$\begin{array}{l} {\text{q}}_{\phi }\left(\text{y}|\text{x}\right)=N\left(y|\mu_{\phi }\left(x\right),\mathrm{diag}\left(\sigma_{\phi }^{2}\left(x\right)\right)\right) \end{array}$$

(2) $$\begin{array}{l} {\text{q}}_{\phi }\left(z|\text{y},\text{x}\right)=N\left(z|\mu_{\phi }\left(y,x\right),\mathrm{diag}\left(\sigma_{\phi }^{2}\left(x\right)\right)\right) \end{array}$$

Where *x* represents a molecule and y represents its continuous valued properties. Given a variable *x*, the properties *y* are predicted as Equation (3):


(3) $$\begin{array}{l} \text{y}\sim N\left(\mu_{\phi }\left(x\right),\mathrm{diag}\left(\sigma_{\phi }^{2}\left(x\right)\right)\right) \end{array}$$

In the molecule generation process, we use the decoder network p_θ_(x ∣ y, z) to generate molecules by the following equation:


(4) $$\begin{array}{l} \hat{\text{x}}=arg\underset{\text{x}}{\max}p_{\theta }\left(\text{x}|\text{y},\text{z}\right) \end{array}$$

### 3.2 Generative model objective

The definition of loss function refers to previous research (Kingma et al., 2014). In this study, the variational lower bound −*L*(*x, y*) of the log-probability of a labeled instance (*x, y*) is showed in Equation (5):


(5) $$\begin{array}{l} \log p(x,y)\geq E_{q_{\phi }(z|x,y)}[\log p_{\theta }(x|y,z)+\log p(y)\\ \;+\log p(z)-\log q_{\phi }(z|x,y)]\\ \;=E_{q_{\phi }(z|x,y)}\left[\log p_{\theta }(x|y,z)\right]+\log p(y)\\ \;-D_{\text{KL}}\left(q_{\phi }(z|x,y)\|p(z)\right)\\ \;=-ℒ(x,y) \end{array}$$

Given the data distributions of labeled ${\tilde{p}}_{l}\left(\text{x},\text{y}\right)$, the loss function is defined as Equation (6):


(6) $$\begin{array}{l} J=\underset{\left(\text{x},\text{y}\right)\sim {\tilde{p}}_{l}}{\sum }L\left(\text{x},\text{y}\right)-\beta \cdot \underset{\left(\text{x},\text{y}\right)\sim {\tilde{p}}_{l}}{\sum }||\text{y}-{𝔼}_{q_{\phi }\left(\text{y}|\text{x}\right)}\left[\text{y}\right]|{|}^{2} \end{array}$$

where the last term is mean squared error for generative learning.

We use the decoder network *p*_θ_(**x** ∣ **y**, **z**) to generate a molecule. A molecule representation $\hat{\text{x}}$ is obtained from **y** and **z** by Equation (4). At each time step j of the decoder, the output **x**^(*j*)^ is predicted by conditioning on all the previous outputs (**x**^(1)^, ..., **x**^(*j*−1)^), **y**, and **z**, because we decompose *p*_θ_(**x** ∣ **y**, **z**) as Equation (7)


(7) $$\begin{array}{l} p_{\theta }\left(\text{x}|\text{y},\text{z}\right)=\underset{j}{\prod }p_{\theta }\left({\text{x}}^{\left(j\right)}|{\text{x}}^{\left(1\right)},...,{\text{x}}^{\left(j-1\right)},\text{y},\text{z}\right) \end{array}$$

### 3.3 Training procedure and evaluation metrics

**Figure 2:**
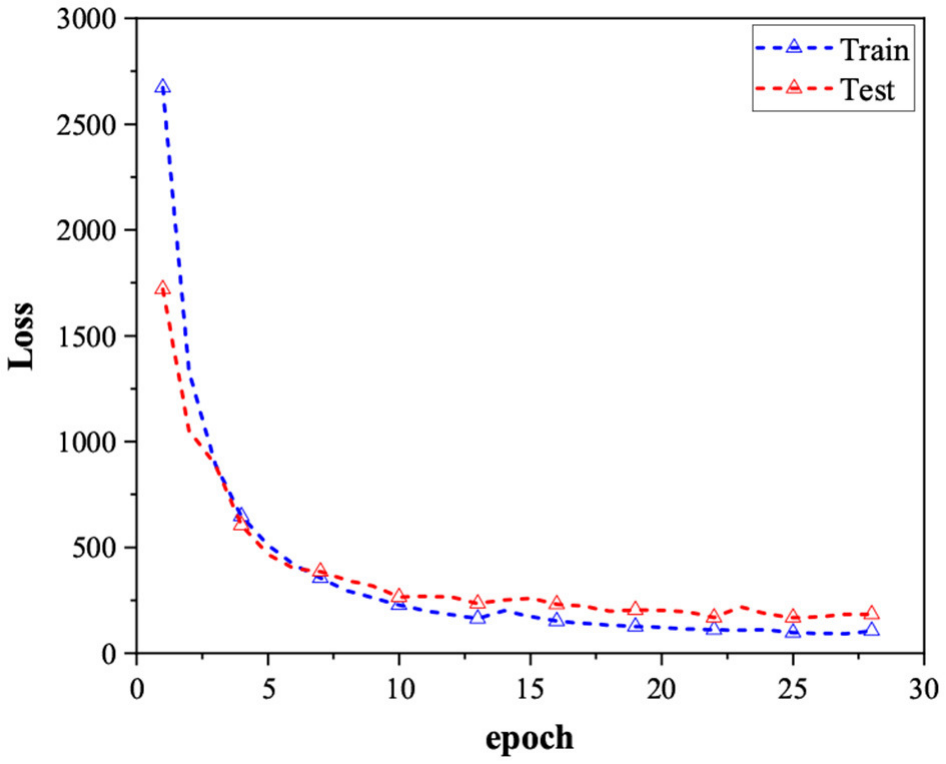
Loss comparison for the training data set and test data set over 30 epochs, where the blue lines indicate the training loss, the red lines indicate the test loss.

The model undergoes training for 300 cycles utilizing the Adam optimizer. To mitigate the risk of overfitting, we employ early stopping during the training process. This means that if the model's performance on the validation set deteriorates compared to the previous cycle, we halt the training and adopt the parameters from the prior iteration as the final outcome. The loss stabilizes after the 25th cycle, as depicted in Figure 2.

The model was implemented using TensorFlow (v2.4.0) in Python (v3.8). We trained it on an NVIDIA 3090 GPU with the learning rate set to 0.001. And the metrics we used are as follows:

• **Validity**: The model's learning capability is evidenced by the rating of authentic molecules among the synthesized compounds as follows (Equation 8).


(8) $$\begin{array}{l} V\text{al}=\frac{\left|V\right|}{n} \end{array}$$

• **Uniqueness**: the percentage of molecules that were really unique when they were generated. Low uniqueness points to recurrent molecule production and a model with little distribution learning as follows (Equation 9).


(9) $$\begin{array}{l} Uni=\frac{\left|\mathrm{set}\left(V\right)\right|}{\left|V\right|} \end{array}$$

• **Novelty**: the percentage of authentically unique molecules that were generated but were not in the training set as follows (Equation 10).


(10) $$\begin{array}{l} Nov=\frac{\left|\mathrm{set}\left(V\right)\cap X\right|}{\left|V\right|} \end{array}$$

Where X is the list of molecules from the provided training set, n is the number of generated samples, and V is the list of created chemically valid molecules.

## 4 Results

In this section, we introduce two publicly available compounds datasets, ZINC and BindingDB, describe the parameters and performance metrics of the evaluation experiments, and evaluate the performance of the proposed method.

### 4.1 Datasets

**Figure 3:**
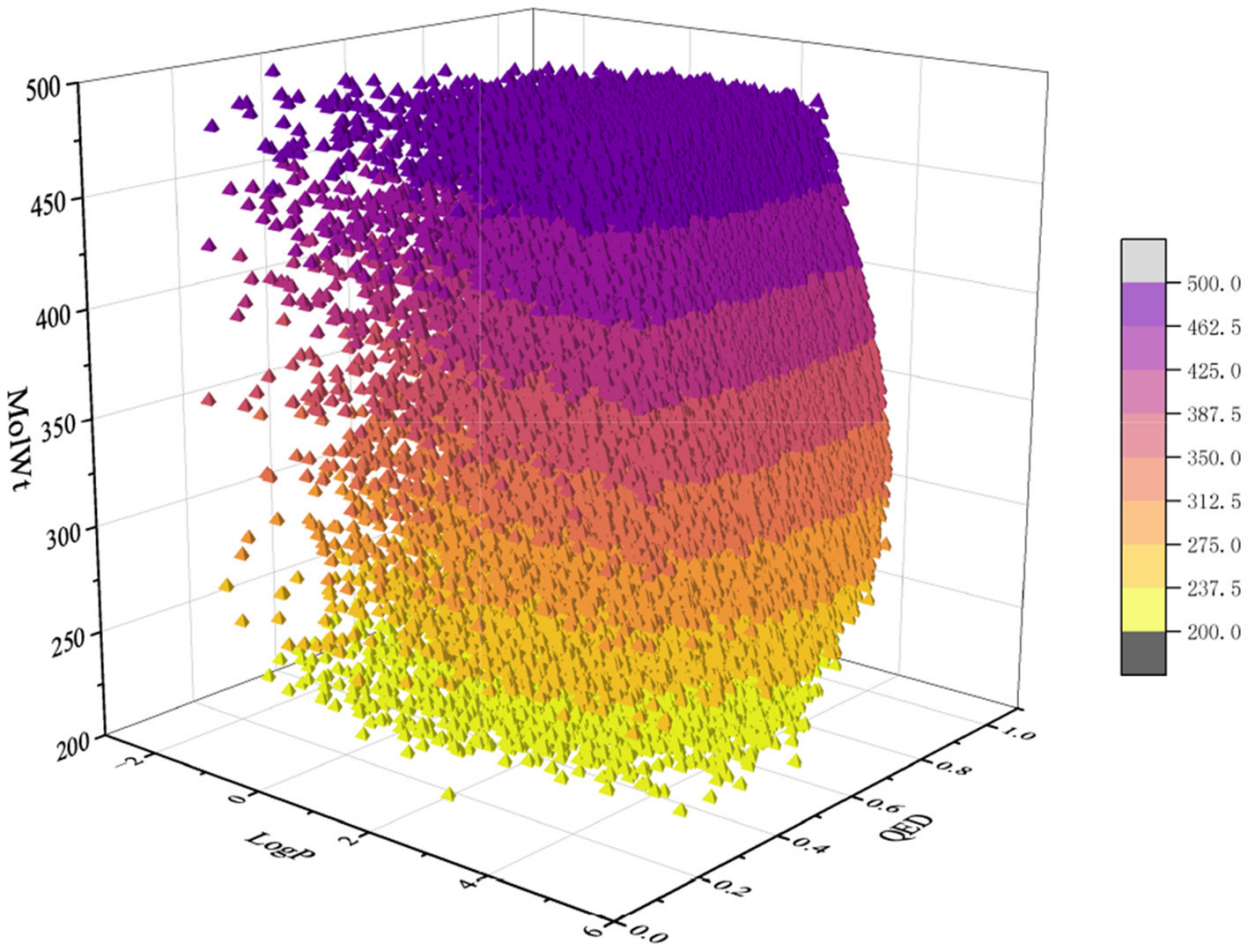
The property distribution of ZINC dataset.

**Figure 4:**
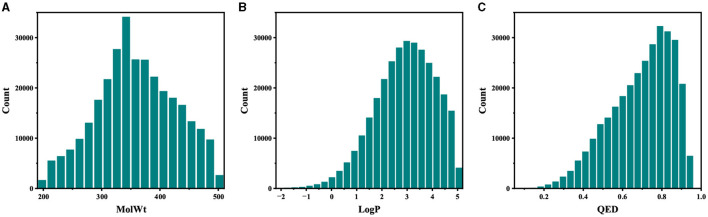
The property distribution of ZINC dataset.

For the pretraining set, we collected 310,000 SMILES strings of drug-like molecules from the ZINC database (Irwin and Shoichet, 2005) with molecular weight (MolWt) ranging from 200 to 500 and logP ranging from 0 to 5. Figures 3, 4 show the larger property distribution of the ZINC database, the larger the property distribution, the stronger the fitting ability of our model. Furthermore, in order to maintain the standardization and unity of data, we used RDkit toolkit (Landrum et al., 2013) to canonicalize the SMILES strings.

We collected molecules with pIC50 or pEC50 greater than 6 as the active molecules of AChE, delete duplicate molecules, and got the fine-tuning sets from Binding DB (Liu et al., 2007). Molecules from SciFinder (Gabrielson, 2018) that fit the aforementioned requirements were added to the fine-tuning set to enlarge it. Finally, 4,996 molecules were obtained and canonicalized using the RDKit toolkit.

**Figure 5:**
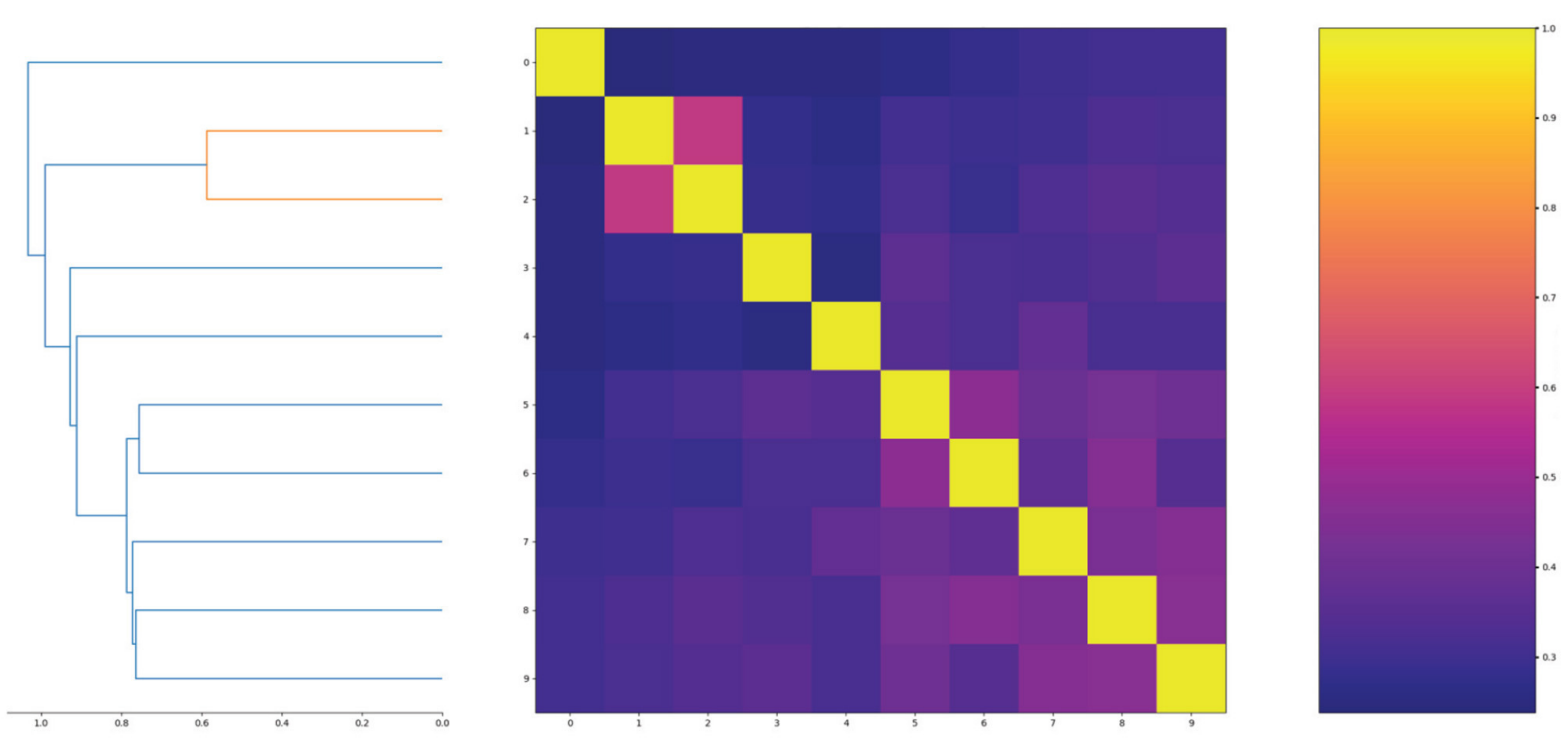
The similarity of randomly selected 10 molecules.

In order to show the diversity of molecules in the data set, we screened molecules with QED (quantitative estimate of drug-likeness) values greater than 8 and logP values between 0 and 3, then randomly selected 10 molecules to calculate molecular similarity. The results are shown in Figure 5.

Some properties of the molecules used in the training process of the model are the following:

- **logP**: The logarithmic value of a substance's partition coefficient in water and n-octane (gasoline). The chemical is more lipophilic the higher the logP value. On the other hand, the more hydrophilic something is, the better its water solubility, and the smaller the logP value.
- **QED**: QED is not based on the properties of chemical structure, but a combination of several molecular properties, which is used to evaluate the drug similarity of molecules. QED quantifies the drug similarity to [0, 1], and the higher the QED score, the higher the drug similarity of molecules.
- **MolWt**: The relative mass of molecules, which refers to the sum of the relative atomic masses of all atoms constituting a molecule. By observing the MW distribution of two groups of molecules, we can check whether the properties of the molecules generated by the model are unbiased or shift toward a certain distribution.
- **SAScore**: Synthesizability score of drug-like molecules based on fragment contribution and complexity penalty.

### 4.2 Performance evaluation

#### 4.2.1 Nonconditioned molecular generation

**Figure 6:**
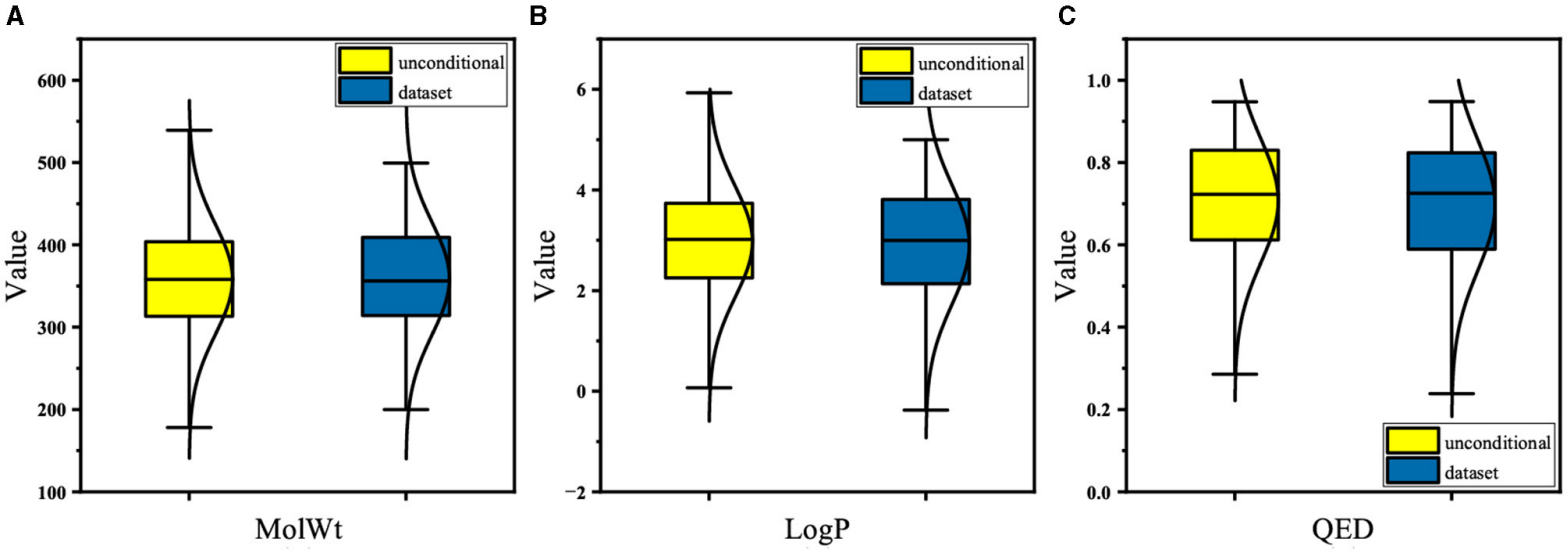
The property distribution of unconditionally generated molecules and ZINC dataset.

In this section, we evaluate the PED's unconditional molecular generation capability and compare it with other molecular generation models. Figure 6 shows the property distribution of unconditionally generated molecules and ZINC dataset, demonstrating how well our model has absorbed the properties of the training set. Moreover, we contrast PED's performance with that of RNN, VAE, and GAN on the ZINC data set. All models use SMILES as input. Table 1 reports the model's performance on the ZINC data set, we show the SMILES and its 2D structure randomly selected from which were generated by the different models simultaneously.

**Table 1:** Comparison of the Different Metrics Corresponding to Nonconditioned Generation of Molecules Using Different Approaches Trained on ZINC Data Set.

**Models**	**Val**.	**Uni**.	**Nov**.
RNN (Grisoni et al., 2020)	0.970	0.999	0.786
VAE (Gómez-Bombarelli et al., 2018)	0.963	0.999	0.532
GAN (Prykhodko et al., 2019)	0.926	0.999	0.921
PED	**0.991**	**1**	**0.906**

The bold values indicates the metric scores of our proposed model.

From the Table 1, PED generates the most reliable and distinctive compounds. However, in the case of novelty, GAN is more likely to generate new molecules. In a nutshell, our model shows the best results in terms of uniqueness and validity, and its novelty is only 0.015 less than the GAN. Therefore, in comparison to other models, PED is the preferable method. Table 2 displays the randomly selected SMILES generated by the PED and other models.

**Table 2:** Randomly selected SMILES generated by the different models.

**Models**	**Sampled SMILES**	**Structure**
RNN (Grisoni et al., 2020)	CN(Cc1ccccc1)C(=O)C1CCCN(S(=O)(=O)c2ccccc2)C1	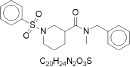
	CCc1ccc(C(=O)NC)nn1	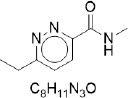
VAE (Gómez-Bombarelli et al., 2018)	O=C(c1ccccc1)N1CCN(c2ccc([N+](=O)[O-])cn2)CC1	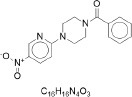
	CCN(CC)S(=O)(=O)c1cccc(C(=O)Nc2ccc(OC(C)C)cc2)c1	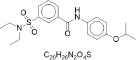
GAN (Prykhodko et al., 2019)	CC1CCCCC12NC(=O)N(CC(=O)Nc1ccccc1C(=O)O)C2=O	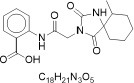
	CCCCCCCCCCCCCCCCCCCCCC1CCC(O)C1(CCC)CCCCCCCCCCCCCCC	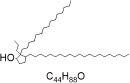
PED	COc1ccccc1NC(=O)CSc1nc(=O)n2ccccc2n1	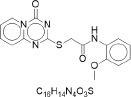
	Cc1ccc(NC(=O)C2CCCN(c3ncnc4onc(C)c34)C2)cc1C	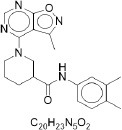

#### 4.2.2 Generation-based on single properties

**Figure 7:**
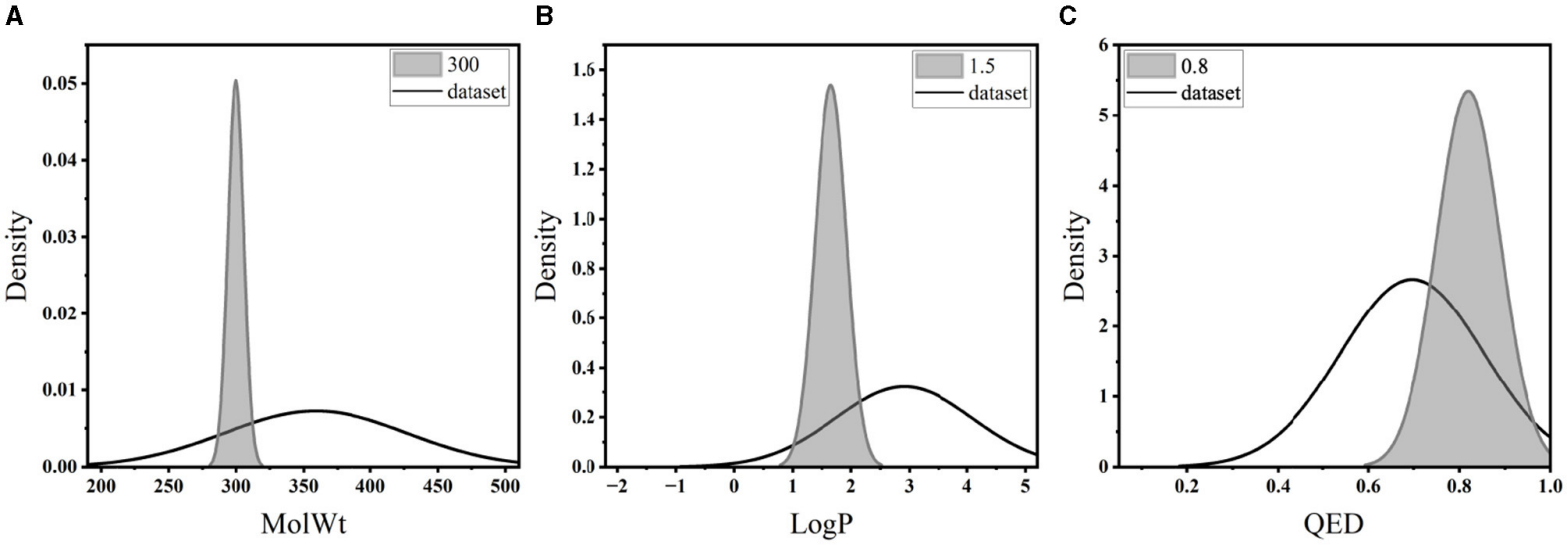
Distributions of properties of generated molecules while controlling a single property.

In this part, we assessed PED's ability to generate molecules with the desired property. We defined the values of LogP, MolWt, and QED accordingly, and created 5,000 molecules under each scenario to evaluate the ability of the model to produce molecules possessing the targeted characteristics. Table 3 illustrates the validity, distinctiveness, and originality scores for each specific condition. PED can still efficiently generate high-quality molecules during the conditional molecular generation process. The property distribution of the generated molecules is shown in Figure 7.

**Table 3:** Comparison of different metrics while generating molecules conditioned on single property based on training on ZINC data set.

**Condition**	**Val**.	**Uni**.	**Nov**.
MolWt	0.985	0.913	1
LogP	0.966	0.951	1
QAE	0.975	0.920	1

Table 3 reveals that when the density of the target value in the training set's distribution was diminished, there was a slight increase in the percentage of inaccurate molecules. Moreover, the model yielded a greater number of replicated molecules when the property forecast for a given condition was notably precise. In particular, the molecules generated by the model did not appear in the training set. In Figure 7, distributions of generated molecules' attributes are shown while optimizing single property, and the distribution is centered around the desired value. The results show that the model can still generate molecules with desired properties without additional optimization steps.

### 4.3 Generating the molecular library for AChE

Acetylcholine (ACh) is the first neurotransmitter discovered by human beings, and its mediated neurotransmission is the basis of nervous system function. Nevertheless, AChE is an efficient hydrolase that catalyzes the hydrolysis of ACh, which regulates the concentration of ACh at synapses and then terminates ACh-mediated neurotransmission. Thus, in this segment, our goal was to create a compound library targeting the AChE receptor. This would aid in the synthesis of newer inhibitors that are not only more potent but also exhibit reduced side effects.

**Figure 8:**
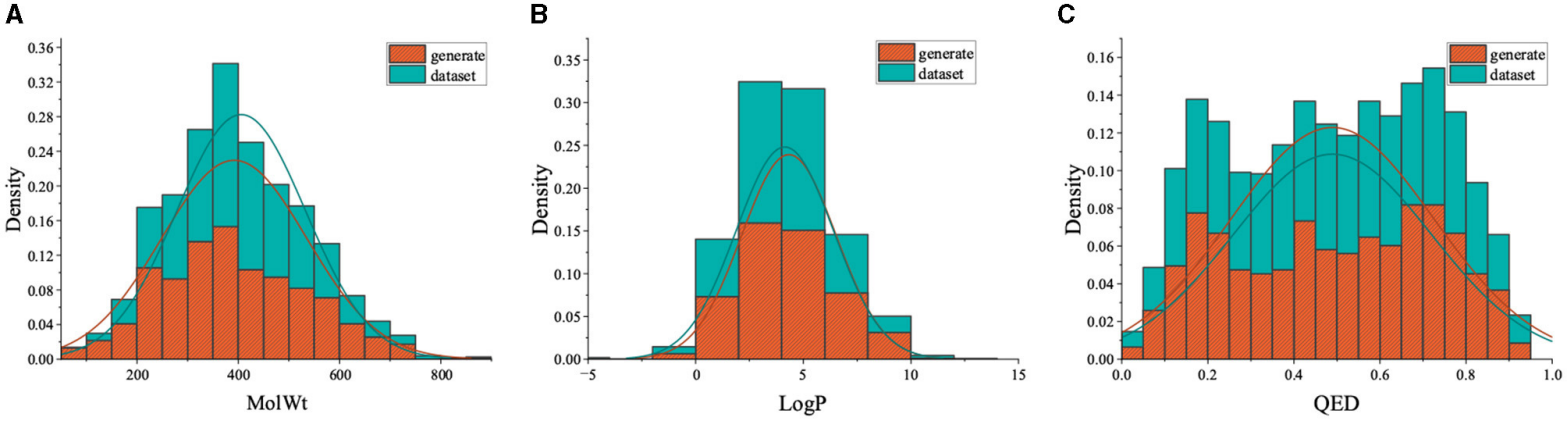
Comparison of property distribution of fine-tuning data set and molecules generated by fine-tuning model.

**Figure 9:**
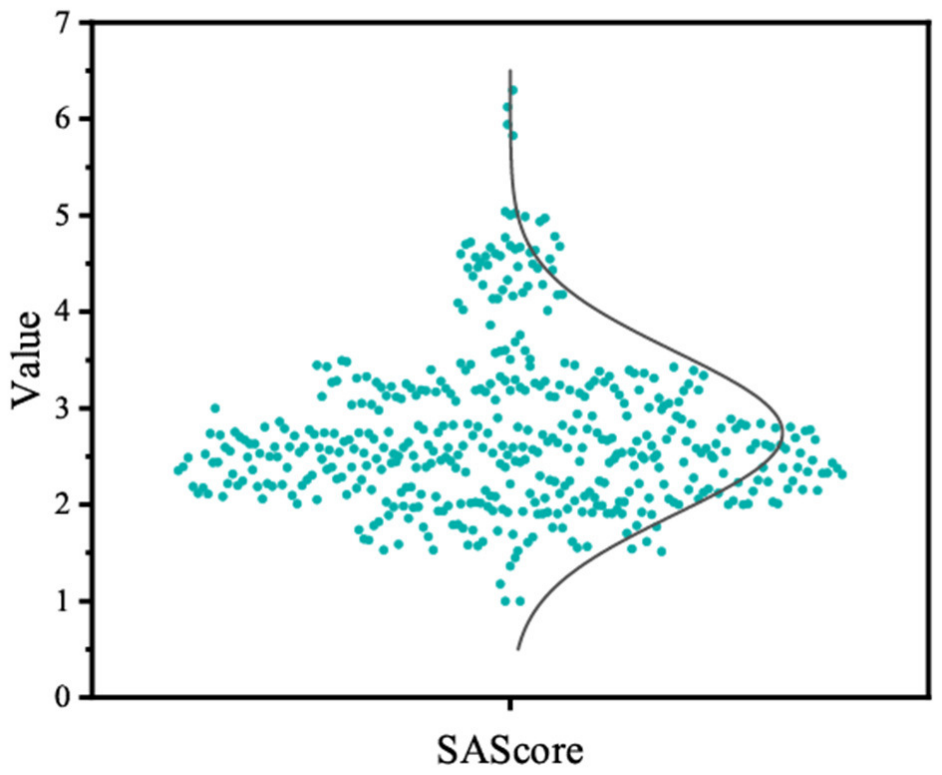
The distribution of SAScore.

We gathered molecules from Binding DB that displayed activity toward AChE receptors to formulate the training dataset. In the end, 4,996 molecules were utilized to constitute the fine-tuning dataset. As depicted in Figure 8, we chose 500 molecules synthesized by the fine-tuned model, which exhibit physicochemical attributes and occupy the chemical space analogous to the fine-tuning set. Additionally, the distributions of QED in the generated molecules are similar to those in the fine-tuning set of compounds. Furthermore, the distribution of SAScore is concentrated between 1 and 5, as shown in Figure 9, indicating that most of the molecules generated are easy to synthesize.

**Figure 10:**
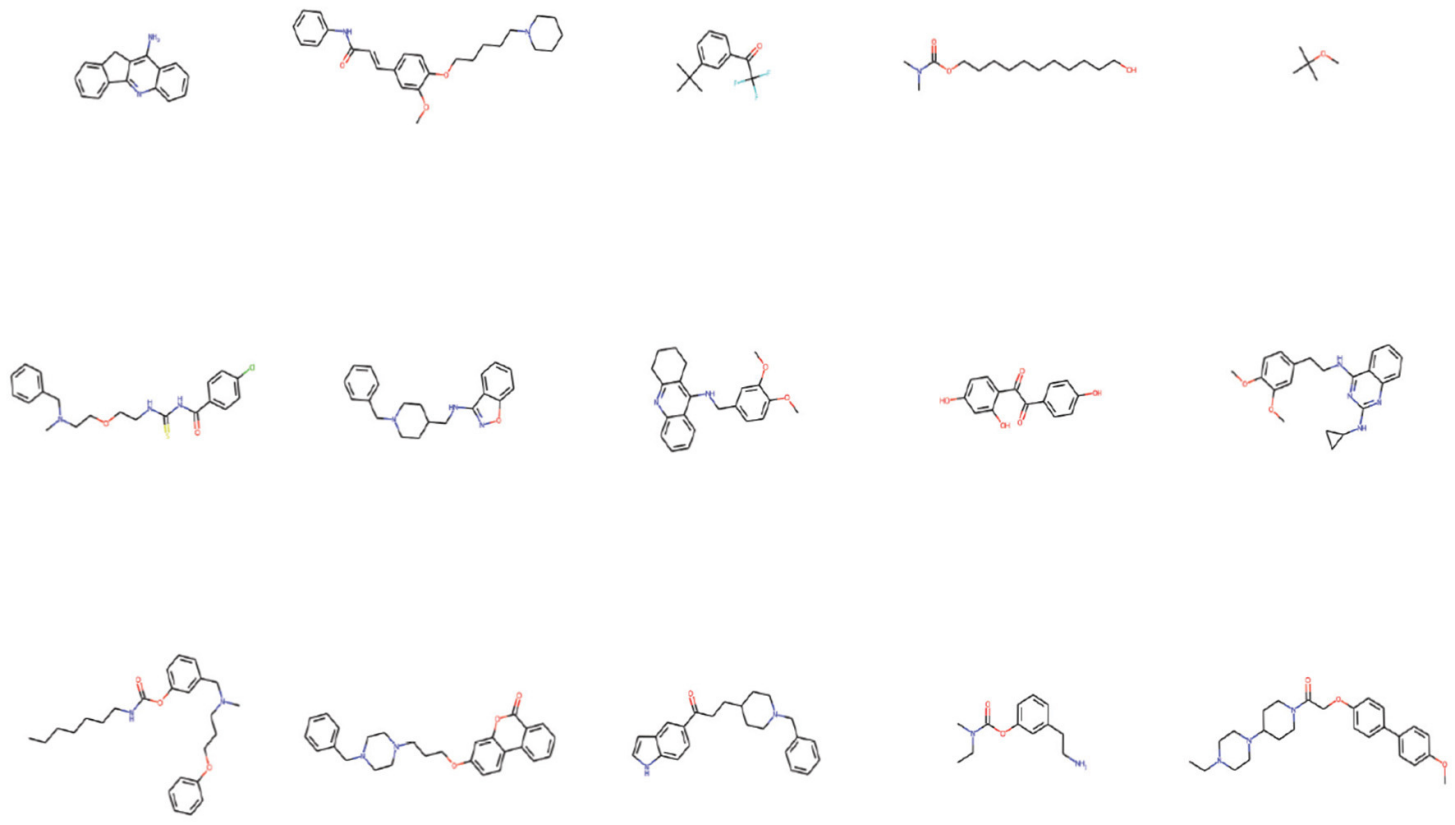
Examples of generated molecules using PED fine-tuning model.

**Figure 11:**
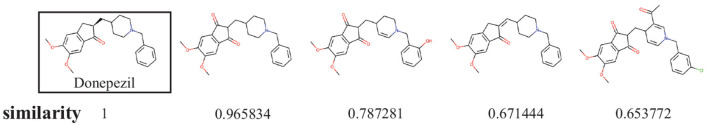
The most similar molecules to Donepezil among the generated molecules.

**Figure 12:**
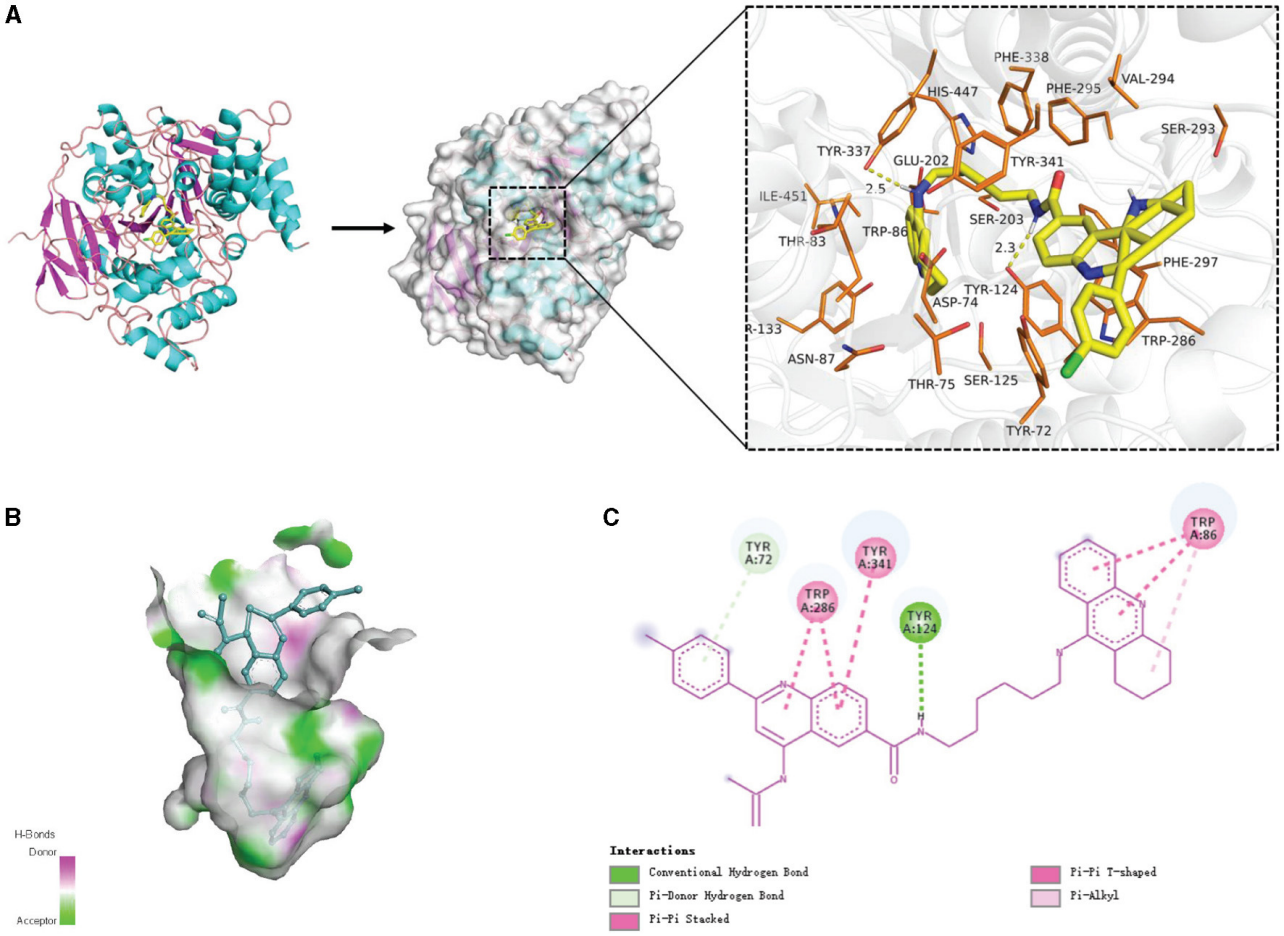
The interaction diagram between the generated molecular and the Acetylcholinesterase (Uniprot ID: P22303). **(A)** Molecular docking and 3D display of the interaction diagram between the generated molecular and the Acetylcholinesterase (Uniprot ID: P22303). **(B)** Hydrogen bond coloring display of the interaction diagram between the generated molecular and the Acetylcholinesterase (Uniprot ID: P22303). **(C)** Local 2D display of the interaction diagram between the generated molecular and the Acetylcholinesterase (Uniprot ID: P22303).

Figure 10 shows examples of the generated molecules. Simultaneously, we compared the molecules with donepezil (Birks and Harvey, 2018) (ID: E20) by ECFP4 (Rogers and Hahn, 2010) similarity method (Figure 11). As shown in the Figure 11, the model can effectively generate molecules similar to the training set. This indicates that our model can generate molecules that are effective for ACHE to a great extent. In order to further verify this conclusion, we conducted molecular docking experiments with Autodock Vina (Trott and Olson, 2010). The ligand in the crystal structure of human acetylcholinesterase in complex with donepezil (Dileep et al., 2022) (PDB ID:7E3H) was removed, then recorded the pocket position simultaneously. Molecules in the molecular library are docked with receptors at the pocket position using Autodock Vina. The docking scores are shown in Table 4, and the results showed that the molecules in the molecular library had high affinity with the target receptor (Figure 12). Based on the molecular docking results, as depicted in Figure 12A, the interaction between the small molecule and the protein primarily involves hydrogen bonding and hydrophobic interactions. Specifically, the N atom of the small molecule forms hydrogen bonds with the hydroxyl O atom of the Tyr124 amino acid residue (Tyr124=O... H- N, 2.3Å), as well as with the hydroxyl O atom of the Tyr337 residue (Tyr337=O... H- N, 2.5Å). Additionally, Figure 12B illustrates that the heteroatoms in the small molecule can engage in hydrogen bonding interactions with the active pocket of the protein, with the distribution of hydrogen bond donors and acceptors shown in Figure 12B. Furthermore, the 2D interaction analysis (Figure 12C) revealed that the hydrophobic carbon chain of the small molecule interacts with the hydrophobic amino acids Thr83, sn87, Trp286, Phe338, and Ile451 of the protein. Moreover, the small molecule forms π−π stacking interactions with the amino acid residues Trp86, Trp286, and Trp341, enhancing its binding affinity to the protein. Given the close proximity between the small molecule and other amino acid residues, it is hypothesized that van der Waals interactions may occur between them.

**Table 4:** Results of the molecular docking.

**Protein**	**Generated SMILES**	**Affinity(kcal/mol)**
Acetylcholinesterase (Uniprot ID:P22303)	O=C(NCCCCCCNc1c2c(nc3ccccc13)CCCC2)c1ccc2nc(-c3ccc(Cl)cc3)c3c(c2c1)NCCC3	-12.9
	O=C(Cc1cc(=O)oc2cc(O)ccc12)NCCCNc1c2c(nc3cc(Cl)ccc13)CCCC2	-12.7
	COc1cc2c(cc1OC)C(=O)C(=Cc1ccc(N3CC[N+](C)(Cc4ccccc4)CC3)cc1)C2	-12.7
	COc1ccc(Cn2cc(C(=O)NCCCNc3c4c(nc5ccccc35)CCCC4)c(=O)c3ccccc32)cc1	-12.6
	COc1ccc(Cn2cc(C(=O)NCCCNc3c4c(nc5cc(Cl)ccc35)CCCC4)c(=O)c3ccccc32)cc1	-12.5

## 5 Discussion

In this work, in order to improve the cognitive technology of AI, we propose a cognitive conditional molecular design model based on VAE to efficiently generate a molecule library with intended properties and screen molecules that can inhibit AChE activity from the library as lead compounds to accelerate the treatment of AD. The model can simultaneously perform both property prediction and molecule generation. We see through our benchmarking experiments that our model shows very high validity, novelty and uniqueness scores for the data sets. Furthermore, the statistics indicate our model's strong control over intended properties for molecular generation under conditional molecular generation. In addition, the statistical data show that our model has a strong ability to control the expected properties of molecular generation under conditional generation. We used AChE active molecule data set to fine-tune the model, generated a molecular library for the target receptor, and ultimately verified the binding ability of molecules to AChE through molecular docking. PED has shown to be promising from a practical and theoretical point of view. It can generate new drug-like molecules for AChE and provide guidance for AD to develop new drugs, which means our model has strong cognitive capacities.

However, there are still some limitations of this work. In this study, we used the traditional strategy to verify the molecular activity, And it is aimed at a single target for molecular generation. Because the occurrence and development of AD involves various complex regulatory networks and changes of regulatory factors, multi-target compounds are the trend of AD drug research and development at present. In the future, we will study a new generation model for multi-target molecular generation and introduce a new deep learning model to predict the activity of generated molecular against target, aiming to automatically generating active molecule library.

## Data availability statement

Publicly available datasets were analyzed in this study. This data can be found here: ZINC database (zinc.docking.org/) and Binding DB (www.bindingdb.org/).

## Author contributions

DL: Conceptualization, Data curation, Methodology, Writing—original draft, Writing—review & editing. TS: Data curation, Funding acquisition, Methodology, Project administration, Resources, Supervision, Visualization, Writing—review & editing. SW: Conceptualization, Data curation, Formal analysis, Funding acquisition, Investigation, Methodology, Supervision, Validation, Visualization, Writing—review & editing. KN: Formal Analysis, Investigation, Resources, Supervision, Validation, Visualization, Writing—review & editing.
